# Perioperative Takotsubo Syndrome Following Breast Prosthesis Explantation and Capsulectomy: A Case Report

**DOI:** 10.1177/22925503261419791

**Published:** 2026-02-15

**Authors:** Akhil Nair, Calandra Li, Basma Bamakhrama, Matthew McRae, Blake Murphy, Mark McRae

**Affiliations:** 1 Temerty Faculty of Medicine, 12366 University of Toronto, Toronto, Ontario, Canada; 2 Division of Plastic and Reconstructive Surgery, 12366 University of Toronto, Toronto, Ontario, Canada; 3 Division of Plastic Surgery, Unity Health, 10071 St. Michael's Hospital, Toronto, Ontario, Canada

**Keywords:** capsulectomy, perioperative cardiomyopathy, takotsubo syndrome, breast implant explantation, capsulectomie, cardiomyopathie périopératoire, explantation de prothèse mammaire, syndrome de Takotsubo

## Abstract

Takotsubo syndrome (TS), a transient cardiomyopathy characterized by left ventricular wall motion abnormalities often mimics presentation of acute coronary syndrome. Although the pathophysiology is not completely clear, it is thought to occur as a result of catecholamine-induced myocardial toxicity. Here we report a case of a 38-year-old female patient who developed perioperative atypical TS during capsulectomy, breast implant explantation, and auto-augmentation. The lessons from management of the severe acute onset event in a rather routine procedure is valuable for raising awareness and should assist with prompt recognition and effective management.

## Résumé

Le syndrome de Takotsubo, une cardiomyopathie transitoire caractérisée par des anomalies du mouvement de la paroi ventriculaire gauche, mime souvent la présentation d’un syndrome coronarien aigu. Bien que la physiopathologie ne soit pas complètement élucidée, on pense qu’il résulte d’une toxicité myocardique induite par les catécholamines. Nous rapportons le cas d’une patiente de 38 ans ayant développé un syndrome de Takotsubo atypique périopératoire lors d’une capsulectomie, d’une explantation de prothèse mammaire et d’une auto-augmentation. Les enseignements tirés de la prise en charge de cet événement aigu et sévère survenu lors d’une intervention relativement routinière sont précieux pour sensibiliser les cliniciens et devraient faciliter la reconnaissance rapide et la prise en charge efficace.

## Introduction

Takotsubo syndrome (TS) first reported in 1990,^1^ acquired its name due to resemblance of a ballooned left ventricle to a Japanese vessel used for capturing octopuses. It mimics acute coronary syndrome (ACS) presenting with acute onset angina, dyspnea, and syncope. Patients may also experience arrhythmias, cardiogenic shock, and asystole^2–4^ along with elevation in cardiac biomarkers, and electrocardiograph (ECG) findings.^5^ The stark similarity of TS to ACS makes it challenging to diagnose especially in a perioperative setting.

TS accounts for 2%-3% of all patients presenting with ACS, while women >50 years accounted for 80% of TS diagnosis.^6,7^ Pathophysiology of TS is not completely understood. The catecholamine theory suggests that a surge in epi/norepinephrine serum levels can cause myocardial hypercontractility and injury.^3^ Two key observations support this theory. One, the histological finding of contraction band necrosis in TS patients which is also seen in pheochromocytoma, a known cause for catecholamine-induced cardiomyopathy.^8,9^ Second, the characteristic left ventricle apical ballooning in TS is attributed to the presence of higher density of β-adrenergic receptors at the heart's apex.^3,8^

Here, we report a case of perioperative TS during bilateral breast prosthesis removal and mastopexy.

## Case Presentation

A 38-year-old female experienced discomfort, pain, and heaviness due to breast implant capsular contracture implanted in 2017. Upon assessment they were a good candidate for bilateral implant explantation and mastopexy. Her past medical history was significant for chronic migraines, joint pain, childhood-onset anxiety disorder, obsessive-compulsive disorder, and anemia. She had no previous history of cardiac concerns and demonstrated good exercise tolerance.

On the day of surgery, preprocedural anesthesia work up was unremarkable and reported cardiovascular parameters within normal limits. At 8:08 AM Cefazolin 2 g, Hydromorphone 0.6 mg IV and Ondansetron 4 mg IV were administered. Propofol 200 mg, Fentanyl 150 mcg, and Rocuronium 50 mg IV were used for general anesthesia induction and maintained via inhaled Sevoflurane.

First, incision was made on the right breast through the skin, subcutaneous tissue, and implant; the flaps were elevated superiorly and inferiorly to access pectoralis major which was subsequently dissected off the implant. 20 cc of 0.25% Bupivacaine-epinephrine mix was administered at 9:18 AM to block pectoralis and intercostal nerves and for prolonged post-operative analgesia. Capsule was circumferentially dissected and removed with the breast implant. The left breast was operated upon in the same sequence. Subsequently, inferior pedicle auto-augmentation was performed under the areola to provide additional projection and improve shape bilaterally. Finally, the nipple-areola complex was repositioned vertically, transverse limb trimmed, and the incisions were closed in standard layered fashion.

At 9:38 AM the patient received Glycopyrrolate 0.4 mg, Neostigmine 2.5 mg, and Esmolol 20 mg intravenously for neuromuscular blockade reversal and hemodynamic stability, immediately, developing transient cardiac arrhythmia with premature atrial and ventricular contractions and supraventricular tachycardia. Blood pressure and pulse were preserved during this episode; rapidly progressing to pulseless ventricular tachycardia and fibrillation. Chest compressions were initiated and lasted for less than a minute before she returned to spontaneous circulation. She was promptly dosed with Magnesium Sulfate 2 g and Calcium Gluconate 1 g IV for ∼5 min starting at 9:40 AM. Once hemodynamically stable, 50 mg Esmolol and 10 mg Metoprolol was administered based on post-arrest ECG findings of sinus tachycardia and conduction blocks before being transferred to post-anesthesia care. Apart from hypotension which was corrected with 1L of IV fluids, she reported no angina, dyspnea, or palpitations. Troponin I at 824 ng/L and an elongated QTc interval (507 ms) indicated myocardial injury due to cardiac strain or transient ischemia during the perioperative episode. Continued measurements of Troponin I (q8h) obtained a peak value of 2260 ng/L.

**Figure 1.**
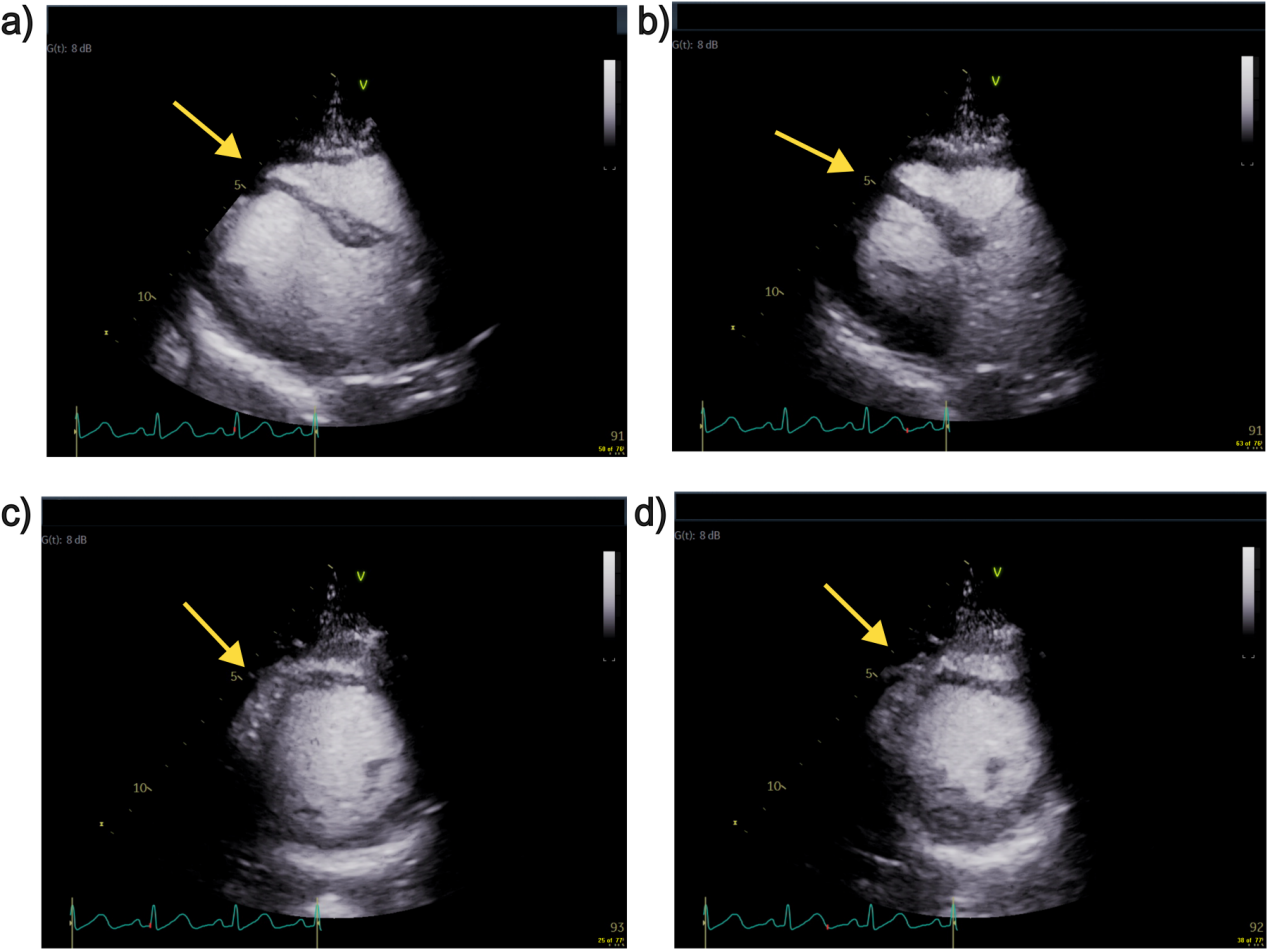
TTE images extracted as freeze frames. (a) Parasternal long axis view of end diastole. (b) Parasternal long axis view of end systole. (c) Parasternal short axis view of end diastole. (d) Parasternal short axis view of end systole. Yellow arrows indicate site of hypokinesis.

On post-operative day (POD)1 transthoracic echocardiogram was performed showing normal left ventricular size, reduced ejection fraction (45%-50% visually), severe mid-distal anteroseptal, and milder inferoseptal hypokinesis (Figure 1). Coronary angiography and left heart catheterization indicated clean coronary arteries and detected the same segmental wall motion abnormalities (WMA). Troponin I while remaining elevated, showed downtrending at 1021 ng/L, and the patient reported no dyspnea, syncope, or angina and was comfortable to take walks around the unit. ECG showed a return to sinus rhythm and QTc interval within normal ranges. On POD 2 and 3, the downward trend in Troponin I levels continued from 1021 ng/L to 152 ng/L. Coronary angiography still indicated no pathology, while WMA persisted consistent with diagnostic criteria for atypical takotsubo stress cardiomyopathy.

Given the abnormal QT dynamics, use of QT prolonging medications, the dramatic QT presentation suspected to be *Torsades de pointes* even though not consistent with monomorphic ventricular tachycardia received the recommendation for implantable cardioverter-defibrillator (ICD) from an electrophysiologist. On POD 9, the patient successfully underwent ICD placement and threshold testing.

At the 2- and 6-week follow-up, the patient continued to have mild discomfort from the placement of the ICD and reduced exercise tolerance, but is overall recovering as expected and reported no further complications.

## Discussion

Our patient presented with mid-ventricular WMA, QTc prolongation (resolved on POD1), elevated troponin, and no evidence for coronary blockages, which combined confirms TS based on Mayo Clinic^10^ and InterTAK diagnostic criteria.^11^ Alternatively, the dramatic presentation of QTc interval prolongation, suspected *Torsade de pointes*, early resolution and return to sinus rhythm, and prolonged use of escitalopram may raise concerns for QTc prolongation to be independent of TS.

Majority recorded cases of TS are in post-menopausal women with rare instances in younger women often induced by excess catecholamines.^12^ It would be difficult to isolate a single precipitating factor here, stressors before the surgery, response to bupivacaine: epinephrine or anesthesia reversal drugs may have all contributed to the cardiac event. Patient's psychiatric diagnoses could have predisposed her to TS, since up to 50% of patients with the diagnosis of TS have a history of psychiatric or neurologic illness^13^—an element incorporated into InterTAK diagnostic risk score.^14^

Timely diagnosis and treatment are paramount to reduce morbidity and initiate early recovery. Full recovery in TS is expected within a few days to weeks and ∼5% of cases experience another episode within 3.8 years.^15^ Progressive downtrending of troponin levels, no angina, dyspnea, palpitations, or syncope and normal sinus rhythms are indicative of improvement in our patient, long-term follow-up was advised to avoid future complications.

In conclusion, TS remains underrecognized in perioperative settings and even rarer within the plastic surgery ORs. Awareness regarding preoperative risk assessment and familiarity to successfully managed cases would help plastic surgeons and anesthesiologists in early detection and effective management of TS.

## Supplemental Material

**Figure:** Video 1. Video transthoracic echocardiogram of parasternal long axis showing septal hypokinesis

**Figure:** Video 2. Video transthoracic echocardiogram of parasternal short axis showing septal hypokinesis
